# Crosstalk analysis of carbon nanotube bundle interconnects

**DOI:** 10.1186/1556-276X-7-138

**Published:** 2012-02-17

**Authors:** Kailiang Zhang, Bo Tian, Xiaosong Zhu, Fang Wang, Jun Wei

**Affiliations:** 1 School of Electronics Information Engineering, Tianjin Key Laboratory of Film Electronic and Communication Devices, Tianjin University of Technology, Tianjin, 300384, China; 2 Singapore Institute of Manufacturing Technology, 71 Nanyang Drive, Jurong, 638075, Singapore

**Keywords:** interconnects, carbon nanotube, bundles, simulation, crosstalk

## Abstract

Carbon nanotube (CNT) has been considered as an ideal interconnect material for replacing copper for future nanoscale IC technology due to its outstanding current carrying capability, thermal conductivity, and mechanical robustness. In this paper, crosstalk problems for single-walled carbon nanotube (SWCNT) bundle interconnects are investigated; the interconnect parameters for SWCNT bundle are calculated first, and then the equivalent circuit has been developed to perform the crosstalk analysis. Based on the simulation results using SPICE simulator, the voltage of the crosstalk-induced glitch can be reduced by decreasing the line length, increasing the spacing between adjacent lines, or increasing the diameter of SWCNT.

## Introduction

Due to electron scattering on copper wire surface and grain boundary, the resistivity of a copper wire will increase rapidly when the interconnect feature size becomes smaller than 45 nm [1]. As a result, the time delay of the transmission signal will increase dramatically, which will restrict the circuit performance. Besides, as the integration density of interconnects increases, crosstalk issues will be the concerns. The crosstalk issue directly affects the circuit performance. To address the issues, carbon nanotube (CNT) interconnects have recently been proposed as ideal substitutes in future interconnect designs [2]. CNT can be metallic or semiconducting [3], depending on their chiralities, and metallic CNTs are the preferred candidates for interconnect applications [4-6].

Although a few studies on the crosstalk noise of CNT-based interconnections have been reported [7,8], the influencing factors are not fully understood. Crosstalk is the unexpected voltage noise interference due to the electromagnetic coupling of adjacent transmission lines when the signal propagates in the transmission lines. It is well known that crosstalk between interconnects may cause signal delay and glitch that may be propagated to the output of a receiver, which can cause a logic error at the output of the receiving device [9]. Therefore, to understand the influencing factors which affect the crosstalk voltage of single-walled carbon nanotube (SWCNT) interconnects and how to decrease them are particularly important.

In this paper, the main factors affecting the crosstalk of SWCNT bundle interconnects were studied, including the influence of the SWCNTs position when their length is fixed, which was proposed for the first time. Firstly, we considered three coupled SWCNT interconnects to form a standard parallel wire architecture over a ground plane by calculating the coupling capacitances between adjacent interconnects; this model was then extended to the SWCNT bundle by calculating the corresponding parameters.

## Methodology

### RLC equivalent circuit parameters of SWCNT

**Figure 1:**
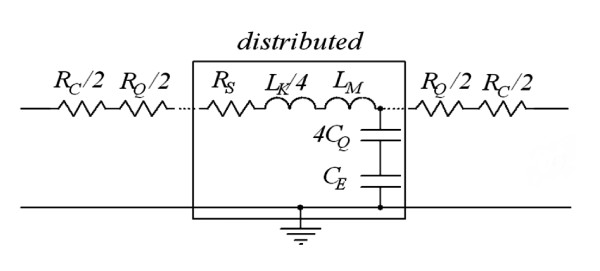
**Equivalent circuit of an individual SWCNT interconnect**.

The equivalent circuit model based on RLC distributed parameters for an individual SWCNT placed away from a ground plane is shown in Figure 1, and its components are explained in detail [10,11] as follows.

The resistance of a SWCNT contains imperfect contact resistance (*R*_C_) which is in the range of 0 to 120 KΩ, quantum resistance (*R*_Q_) (*R*_Q _= *h*/4*e*^2^, and scattering resistance (*R*_S_) per unit length (*R*_S _= *h*/(4*e*^2^·*λ*_CNT_)), where *h *is Planck's constant, *e *is the charge of an electron, and *λ*_CNT _is the mean free path length.

The capacitance of a SWCNT includes electrostatic capacitance (*C*_E_) and quantum capacitance (*C*_Q_); the expressions are given by

(1) $$C_{\text{E}}=2\pi \varepsilon /\text{ln}\left(y/D\right)$$

and

(2) $$C_{\text{Q}}=2e^{2}/hv_{\text{F}},$$

where *D *is the diameter, *y *is the distance away from a ground plane treating the CNT as a thin wire, and *v*_F _is the Fermi velocity.

The inductance of a SWCNT includes kinetic inductance (*L*_K_) and magnetic inductance (*L*_M_); the expressions are given by

(3) $$L_{\text{k}}=\frac{h}{2e^{2}v_{\text{F}}}$$

and

(4) $$L_{\text{M}}=\frac{\mu }{2\pi }\ln(\frac{y}{D}).$$

For *D *= 1 nm and *y *= 1 μm, *L*_M _≈ 1.5 pH/μm. Clearly, the magnetic inductance can be neglected.

### Crosstalk modeling for CNT bundle interconnects

In practice, CNT bundles are closer to actual application than individual CNT. Here, the crosstalk modeling is being established.

**Figure 2:**
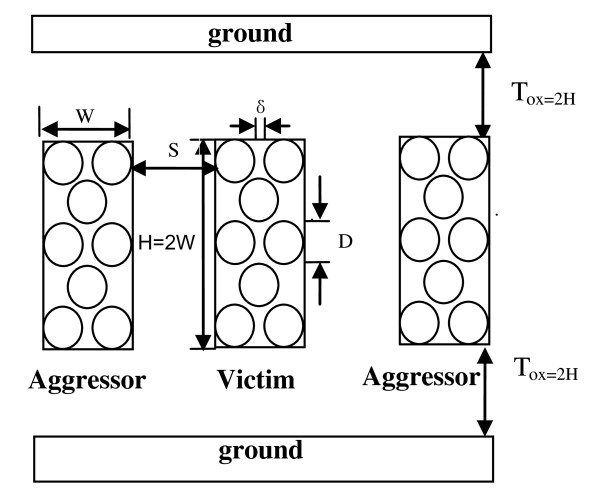
**Cross-sectional view of the interconnect architecture using SWCNT bundle**.

Figure 2 shows the cross-sectional view of the interconnect architecture for further characterization of crosstalk effects in SWCNT bundles. The middle signal wire is filled with CNTs, and it is the same as the adjacent interconnects on both sides of the signal. The width and height of the interconnects are *W *and *H*, respectively. The spacing between two interlayer interconnects is *S*, and the thickness of the interlayer-dielectric is *T*_ox_. *H *= 2W and *δ *≈ 0.34 nm, which is the van der Waals gap.

The total number of SWCNTs (*N*_CNT_) in the bundle is given as

(5) $$N_{\text{CNT}}=N_{\text{W}}\cdot N_{\text{H}}-\text{In}\left[\frac{N_{\text{H}}}{2}\right]$$

where

(6) $$N_{\text{W}}=\text{In}\left[\frac{W-D}{D+\delta }\right]+1$$

and

(7) $$N_{\text{H}}=\text{In}\left[\frac{H-D}{\left(\sqrt{3}/2\right)\left(D+\delta \right)}\right]+1,$$

where *N*_H _is the number of rows in the interconnect bundle, *N*_W _is the number of columns, and *N*_CNT _is the total number of CNTs. Since a SWCNT bundle consists of several individual SWCNT in parallel, the formulas of the resistance, inductance, and capacitance of a SWCNT bundle have been listed in previous papers [12].

**Figure 3:**
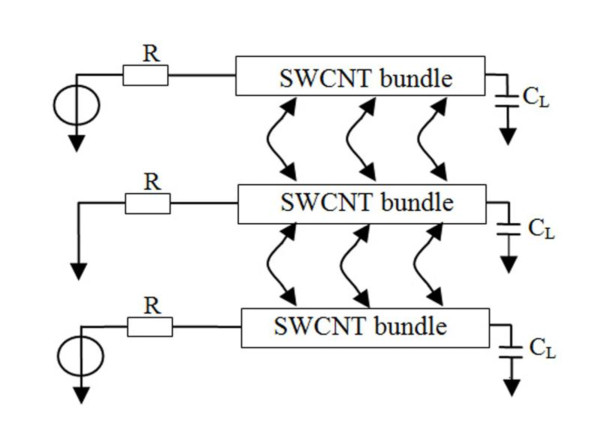
**Geometry of three parallel SWCNT bundle interconnect**.

In order to analyze the influencing factors which affect the crosstalk voltage on adjacent wires, we consider the geometry of three parallel SWCNT bundle interconnects as shown in Figure 3. The wire affected by the crosstalk is termed as victim wire, and the wires that cause crosstalk on the victim wire are termed as aggressor wires. The driver and load parameters are also shown here, where *R *is the equivalent output resistance, and *C*_L _is the input capacitance. To analyze the situation in which there is the largest crosstalk pulse, assume that there is no voltage in the signal line, and the input excitation of the aggressor lines on each side are square wave signal of 1 V with simultaneous switching in the same direction. All geometrical parameters in the simulation below are based on the International Technology Roadmap for Semiconductors (ITRS) 2007 [13].

## Results and discussion

The crosstalk voltage in a SWCNT bundle depends on several factors, such as line length, the position when the length is fixed, spacing between SWCNTs, etc., which will be discussed using the RLC model, respectively. Simulations are performed using SPICE simulator.

**Figure 4:**
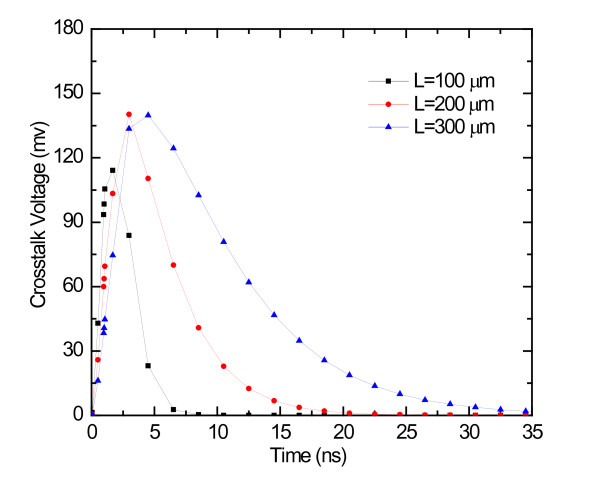
**The effects of length on the crosstalk noise for the 14-nm technology node**.

The crosstalk voltage induced on the victim line by the aggressors for several values of line length for the 14-nm technology node is shown in Figure 4. It can be seen obviously that the peak amplitude and duration increase by increasing the line length. For higher values of length, the crosstalk amplitude keeps a level slightly below 0.15 V, while increase in duration is still applicable. Therefore, for this case, sampling elements with a threshold voltage *V*_DD_/2 = 0.5 V, the induced peak voltage will never reach the threshold voltage, so the induced peak voltage will not produce a logic error at the output of the receiver. Besides, we can reduce the duration time by decreasing the length.

**Figure 5:**
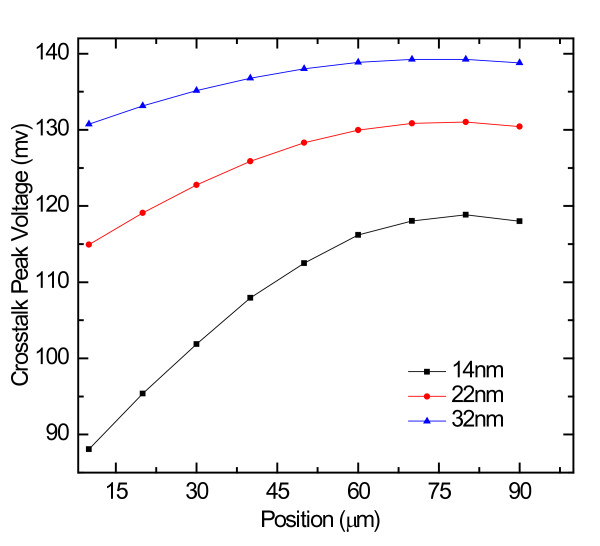
**The effects of position on the crosstalk noise when length is 100 μm**.

The crosstalk peak voltage of SWCNT bundle interconnects induced on the victim line of different positions for the length fixed as 100 μm is shown in Figure 5. As we can see, with an increase in position, crosstalk peak voltage will increase accordingly. But after the position value reaches about 60 μm, it is almost saturated, which indicates that, if there are adjacent interconnections, it is better to place them at the position where there is small crosstalk when they are designed to reduce the impact of crosstalk. In addition, we can conclude that, with the decrease of technology node, crosstalk peak voltage at the same position of a fixed length will decrease correspondingly. That is to say, with the decreasing of feature size, crosstalk caused by the different positions will be reduced as a whole.

**Figure 6:**
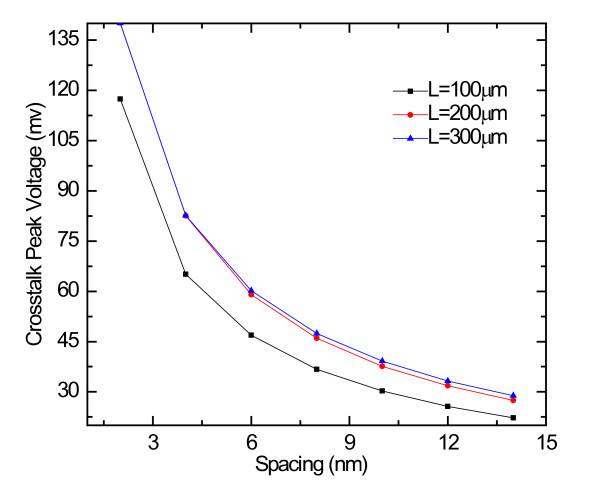
**The effects of spacing on the crosstalk noise for the 14-nm technology node**.

The peak voltages induced by the crosstalk versus the spacing are shown in Figure 6. The figure indicates that the induced peak voltages decrease rapidly as the spacing values increase. This is due to the fact that, with an increase in the spacing, the coupling capacitance reduces rapidly. Therefore, the peak voltages due to crosstalk can be significantly reduced by properly setting the spacing between adjacent lines (e.g., from 2 to 8 nm in this case), but beyond this point, noise reductions will saturate. In addition, as feature size becomes smaller and smaller, line spacing cannot be increased infinitely to decrease crosstalk noise, which needs to set spacing properly.

**Figure 7:**
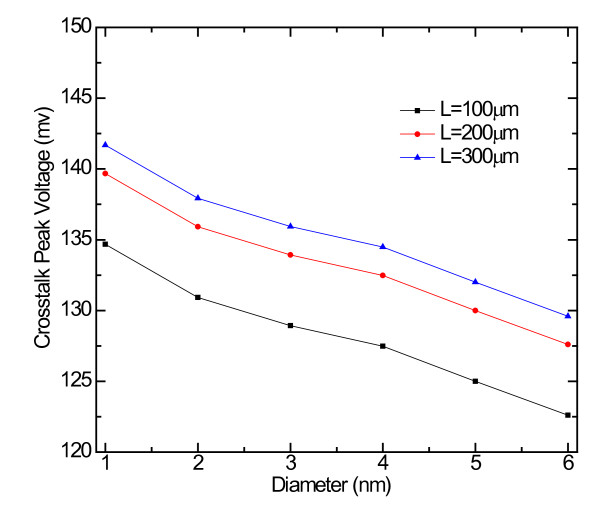
**The effects of diameter on the crosstalk noise for the 14-nm technology node**.

Figure 7 shows the effects of diameter on the crosstalk noise. The crosstalk peak voltage decreases gradually with the increase of the SWCNT diameter for the technology node of 14 nm. When the feature size is fixed, the larger the diameter, the smaller is the number of SWCNT. The voltage amplitude variation value is about 20 mV when the diameter changes from 1 to 6 nm. We can conclude that crosstalk voltage can be reduced by increasing the diameter.

**Figure 8:**
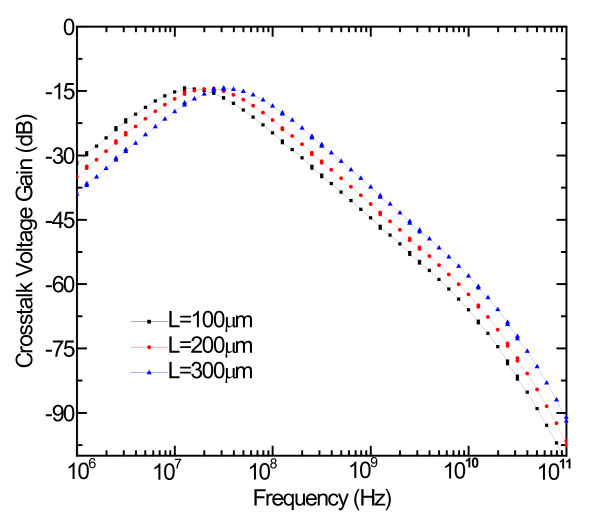
**The effects of frequency on the crosstalk noise for the 14-nm technology node**.

Figure 8 shows the crosstalk voltage gain with the frequency change. The main effect is a spread of the curves on the interconnects. It is worthy to note that, despite the length of the interconnects, the crosstalk waveforms show a relative spacing that is almost constant. With increasing the frequency of the aggressor wires, crosstalk voltage first increases and then decreases after it reaches the peak. When the frequency exceeds 1 GHz, crosstalk voltage decreases gradually with the increase of frequency. Therefore, the peak voltage gain due to crosstalk can be significantly reduced by properly setting the frequency.

## Conclusions

The crosstalk problems of using SWCNT bundle as an interconnect candidate in the future design of integrated circuits have been explored in this paper. Equivalent distributed circuit parameter models of SWCNT bundle are obtained firstly, and then crosstalk issues about parallel SWCNT bundle interconnects are analyzed based on ITRS. The simulations show that significant reduction in crosstalk noise can be achieved by decreasing line length, setting the appropriate position when the length is fixed, increasing spacing between adjacent lines, increasing the diameter of SWCNT as well as selecting the appropriate frequency.

## Abbreviations

CNT: carbon nanotube; SWCNT: single-walled carbon nanotube.

## Competing interests

The authors declare that they have no competing interests.

## Authors' contributions

KZ initiated the idea. BT and XZ drafted the manuscript. FW and JW participated in its design and helped draft the manuscript. All authors read and approved the final manuscript.
